# Prescription of Mood Stabilizers During Pregnancy: A Literature Review

**DOI:** 10.1192/j.eurpsy.2026.12010

**Published:** 2026-07-31

**Authors:** H. Hsine, M. El Behi, A. Bouaziz, S. Kolsi, W. Abbes

**Affiliations:** 1 Psychiatry, La Metairie Clinic, Nyon, Switzerland; 2 Psychiatry, University Hospital of Gabès, Gabes, Tunisia

## Abstract

**Introduction:**

The frequency of bipolar disorder, its early onset, and the requirement for long-term mood stabilizer treatment highlight the challenge of managing bipolar disorder during pregnancy, particularly due to teratogenic risks.

**Objectives:**

The objective of this work is to provide an updated review of the literature, easily applicable by healthcare professionals involved in the follow-up or prescription of mood stabilizers during pregnancy. The aims is to deliver clear information on the risks associated with exposure to these medications and to guide therapeutic decisions, while emphasizing the importance of adherence, as discontinuation could lead to relapse with potentially severe consequences.

**Methods:**

A systematic literature review was conducted, following PRISMA-P methodology. Inclusion criteria were clinical trials and randomized controlled trials published between 1975 and 2024, whose primary objective was to study the effects of exposure to mood stabilizers during pregnancy and to assess teratogenicity.

**Results:**

Our study included 48 articles on the teratogenicity of mood stabilizers during pregnancy.

The teratogenicity of sodium valproate is well known, particularly neural tube defects (spina bifida). This risk is significantly higher compared to other mood stabilizers. More recently, a considerable risk of developmental delays and autism spectrum disorders has been demonstrated in children exposed in utero.

Our review showed that lithium intake during pregnancy is associated with an increased incidence of cardiac malformations when prescribed during the first 50 days of gestation, with Ebstein’s anomaly being overrepresented.

Carbamazepine use increases the frequency of congenital malformations. The most frequently reported include cleft lip and/or palate, cardiac malformations, hypospadias, hypoplasia of distal phalanges and nails, microcephaly, hypertelorism, intrauterine growth restriction, and psychomotor delay. Carbamazepine significantly increases the risk of neural tube defects, particularly spina bifida.

Lamotrigine is the mood stabilizer considered the least concerning for pregnant women, as current evidence on malformative and neurodevelopmental outcomes is relatively reassuring.

Published studies on exposure to atypical antipsychotics (olanzapine, aripiprazole, risperidone, quetiapine) during pregnancy do not show an increased risk of spontaneous abortion, stillbirth, prematurity, or congenital malformations. This provides some reassurance in cases of accidental pregnancy among women already treated with these drugs.

**Conclusions:**

Pregnancy in women with bipolar disorder requires balancing fetal risks and maternal therapeutic success. Management during this period is a challenge, aiming to minimize fetal consequences while preventing maternal relapse due to mood stabilizer discontinuation.

**Disclosure of Interest:**

None Declared

